# Infra-His Block in a Normal Heart

**Published:** 2006-07-01

**Authors:** Mohammad Alasti, Abolfath Alizadeh, Amirfarjam Fazelifar, Abolfath Mohammad Ali

**Affiliations:** Department of Pacemaker and Electrophysiology, Rajaie Cardiovascular Medical Center, Vali-asr Avenue, Tehran, IRAN

**Keywords:** His-purkinje system, infra-his block

A 55 year old man with history of palpitation was referred for electrophysiologic study. Baseline ECG, physical examination and transthoracic echocardiographic study  were normal. Electrophysiologic study revealed normal AH and HV intervals. Pacing of right atrium with a cycle length of 300 msec showed  2:1 AV block. AH interval was 252 msec and the block was infra-his (Figure 1). With continual of right atrial pacing, one to one AV conduction with increasing AH interval to 282 msec and QRS widening (LBBB pattern) were being observed.  HV intervals during 2:1 block and during 1:1 AV conduction were normal.  What is the mechanism? Is it an abnormal finding in this patient?

During 2:1 AV conduction, AH interval was 252 msec and conducting system below His was in absolute refractory period so the impulse could not propagate and infra-his block occurred. After increasing of AH interval to 282, the conducting system below His had enough time to enter the relative refractory period. So AH conduction delay permitted transition from 2:1 AV conduction to 1:1 AV conduction. Infra-his block can be  produced at paced cycle lengths of less than 400 msec in normal His-purkinje system and is not considered abnormal in this case.

## Figures and Tables

**Figure 1:**
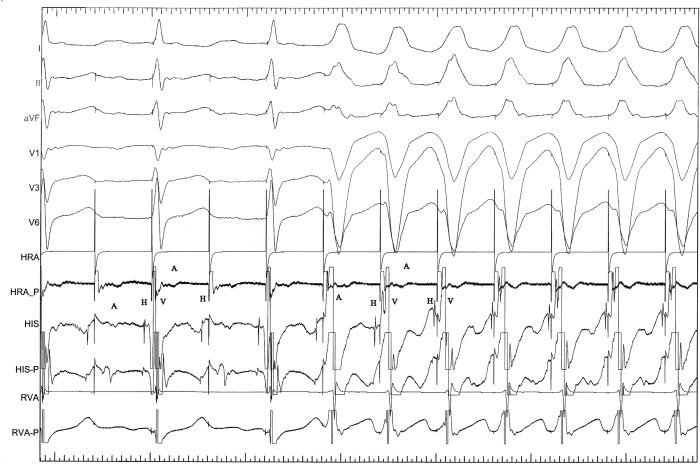
Shows sinus bradycardia at a rate of 52 beats per minute, PR interval of 230 milliseconds and a left bundle branch block.

